# Development and Evaluation of an Adaptive Multi-DOF Finger with Mechanical-Sensor Integrated for Prosthetic Hand

**DOI:** 10.3390/mi12010033

**Published:** 2020-12-30

**Authors:** Changcheng Wu, Tianci Song, Zilong Wu, Qingqing Cao, Fei Fei, Dehua Yang, Baoguo Xu, Aiguo Song

**Affiliations:** 1 College of Automation Engineering, Nanjing University of Aeronautics and Astronautics, Nanjing 211100, China; tommy179931@163.com (T.S.); wuzilg@163.com (Z.W.); fei.fei@nuaa.edu.cn (F.F.); dhyang@nuaa.edu.cn (D.Y.); 2 School of Instrument Science and Engineering, Southeast University, Nanjing 210096, China; xubaoguo@seu.edu.cn (B.X.); a.g.song@seu.edu.cn (A.S.); 3 School of Aviation Engineering, Nanjing Vocational University of Industry Technology, Nanjing 210023, China; caoqingqing87@gmail.com

**Keywords:** prosthetic hand, adaptive grasping, joint angle measurement

## Abstract

To realize the adaptive grasping of objects with diverse shapes and to capture the joint angles of the finger, a multi degree of freedom (DOF) adaptive finger for prosthetic hand is proposed in this paper. The fingers are designed with three joints. The maximum rotation angle of the finger joints is 90°. The angle at which the finger joints bend can be captured. Firstly, the prototype design, forward kinematics and force analysis of phalanges are described in detail. In order to achieve an adaptive motion pattern similar to that of the human hand, this paper investigates the optimization of the torsion spring stiffness coefficient so that the metacarpophalangeal (MCP) joints, proximal interphalangeal (PIP) joints, and distal interphalangeal (DIP) joints of the bionic finger meet a motion ratio of approximately 3:3:1. Then, in order to realize the joint angle measurement in the process of grasping an object, the mechanical-sensor integrated finger joint is designed, and the composition, angle measurement principle and measurement circuit are introduced in detail. Finally, joint angle measurement, movement law evaluation and object grasping experiments are performed to verify the validity of the designed finger. The experimental results show that the root-mean-square (RMS) of the DIP, PIP and MCP angle measurement errors are 0.36°, 0.59° and 0.32°, respectively. The designed finger is able to grasp objects with different shapes stably.

## 1. Introduction

Robotic hands have a wide range of applications. There are many types of robot hands, such as Schunk hand, HERI II hand, Sensor Hand, MPH-II hand, iLimb, etc [1,2,3,4,5,6,7,8]. Some of them are used for assembly into robots, while some of them, prosthetic hands, are used for attachment to the human body to replace the lost limb of an amputee. In patients with upper limb amputation, prosthetic hands can not only cosmetically re-place the lost limb but also restore certain functions of the hand. Lots of researches have been done in this area.

Structure design, information perception and motion control are three important aspects of the research of a prosthetic hand. In terms of structural design, Zhang designed a robotic hand, which has five fingers, each with 2 degree of freedom (DOF) [9]. Li developed a robot hand with underactuated fingers driven by three linkages [10]. Liu proposed an underactuated two finger gripper for grasping unknown objects [11]. Matheus F. Reis proposed a robot hand with three fingers based on elastic tendon linkage for adaptive grasping [12]. Panipat Wattanasiri proposed a prosthetic hand that can perform multiple grip patterns using only one actuator based on linkage drive. However, the hand lacks perception abilities [13]. F.Cordella developed a bionic hand named IH2 Azzurra which has the ability to perceptive, such as detecting the sliding of objects in real time [14]. However, the finger structure is cumbersome due to the integration of a large number of sensing units. The University of Bologna investigated an innovative robotic hand called the DEXMART Hand which has almost all the degrees of freedom of a human hand [15,16]. However, due to the higher number of degrees of freedom, it is more complex to control. In summary, these dexterous hands are either overly complex in structure or lack of features required for dexterous hands.

In terms of information perception, researchers are most interested in the grasp force and information about objects sliding on the hand. Wu investigated a mechanical-sensor integrated finger that aims to measure both grasp force and contact position simultaneously as the hand grasps an object [17]. Based on the principle of surface texture recognition, Venter developed a tactile sensor to detect objects sliding in a robotic hand [18]. Luberto proposed an adaptive grasping strategy based on the object location estimation through point-cloud data [19]. Wookeun developed a finger motion capture system based on soft sensors. And a camera-based motion capture system was used to verify the performance [20]. Othman employed a rotary potentiometer to measure and monitor finger flexion [21].

Multi-DOF is an important characteristic of prosthetic hands. The human hand has 21 degrees of freedom and a unique structure. It is very difficult to design a bionic prosthetic hand that has the same degrees of freedom as a human hand. In practice, the prosthetic hand only needs enough DOFs to be able to perform the most commonly used gestures in daily life. The ability to perceive information is another important characteristic of the prosthetic hand [8,22,23]. For fingers based on rigid linkage, it is not necessary to install angle sensors in the fingers, as the angles of each joint can be calculated by using the relationship between linkages. However, angle sensors are es-sential for a multi-DOF adaptive finger. In the existing studies, most prosthetic hands do not have joint-angle sensors because of the limitation of installation control. This is not conducive to the control system perceiving the grasp state of the artificial hand on an object.

In this paper, a multi-DOF adaptive finger with a mechanical sensor integrated for prosthetic hands is investigated. The finger has the ability to stably grasp objects with diverse shapes and the capability to measure the angle of finger joints.

### Novelty and Contribution

Unlike other bionic finger designs discussed previously, this study proposes a multi-DOF adaptive finger with mechanical-sensor integrated that can stably grasp objects of different shapes and detect the angles of finger joints. The key contributions are listed as follows. Firstly, the forward kinematics analysis is performed on the basis of the prototype design. Subsequently, for the purpose of mimicking the movement of the human hand, a set of spring parameters that can make the angles of three finger joints approximately satisfy the 3:3:1 relationship during the free movement are obtained by analyzing the force on the finger. Lastly, a mechanical sensor-integrated finger joint is proposed in order to achieve accurate measurement of the finger joint angle.

The rest of the paper is organized as follows. Section 2 describes the design of the adaptive finger in detail. The experiments are performed in Section 3, and the conclusions of the paper are in Section 4.

## 2. Design of the Adaptive and Mechanical-Sensor Integrated Finger

### 2.1. Design of the Adaptive Finger Structure

#### 2.1.1. Prototype Design

**Figure 1:**
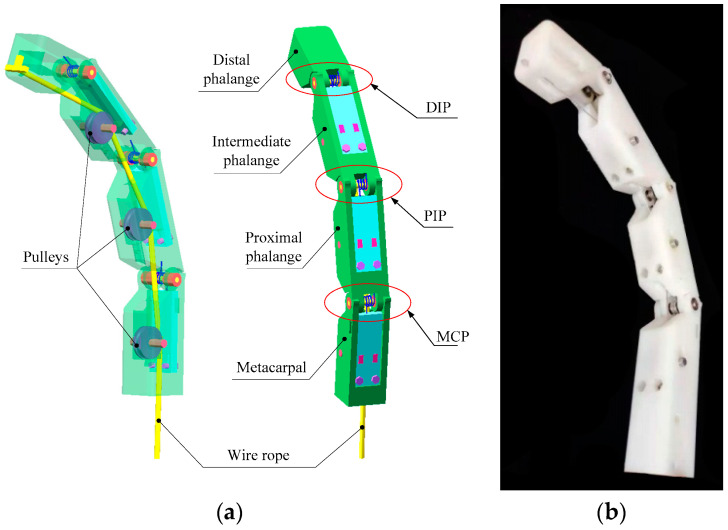
The 3-D model and prototype of the designed finger. (**a**) 3-D model of the designed finger; (**b**) Prototype of the designed finger.

For the human hand, except the thumb, every finger consists of three phalanges: the distal phalange, the intermediate phalange and the proximal phalange. So, we designed the finger with three phalanges and three rotatable joints. The designed finger is driven by a steering gear. There is a wire rope running through three phalanxes. One side of the wire rope is fixed on the top of the distal phalange, and the other side is connected to the swing arm of the steering gear. Therefore, the steering gear rotation drives the wire rope movement to achieve finger bending. The rotatable joint consists of a roller, a torsion spring, and four bearings. The roller passes through the four bearings and the torsion spring. In order to reduce the friction between the wire rope and the phalanges, three guide pulleys are fitted up on the middle finger bone, the proximal finger bone and the metacarpal bone. The fingers are naturally straight in this structure when there is no driving force from the wire rope. Under the driving force of the wire rope, the finger is bent. However, the finger stays straight again when the driving force disappears. In the case that the fingers do not touch the object, the three finger bones move synchronously under the traction of the wire rope. In the process of grasping the object, the movement of each phalange in the finger will not change when the finger with rigid connecting rod structure contacts with an object. As a result, when grasping an irregular object, some phalanges cannot make good contact with the object, which reduces the stability of grasping. For the finger proposed in this paper, the movement of the phalange which contacted with object will be restricted. While in front of the restricted phalanges, the phalanges that are not in contact with the object remain unchanged. It is because of this movement law that the designed finger can well adapt to objects with different shapes and carry out steady grasping. In other words, the elastic-spring finger joints between phalanges can make the maximized contact area with objects during the bending movement of the finger, since each phalange can move freely even other phalanges are restricted by the edge of object. So the adaptive situation is mainly decided by the contacting between finger and object during the grasping process. In this paper, the phalanges are fabricated using 3D printing technology. Figure 1 shows the 3-D model and prototype of the finger.

#### 2.1.2. Forward Kinematics

**Figure 2:**
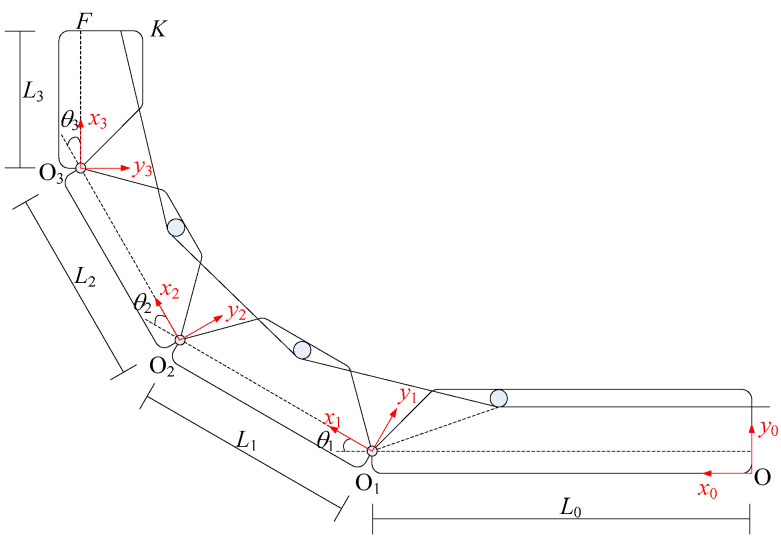
Geometric representation of the finger model.

Figure 2 illustrates the geometric representation. The distal interphalangeal (DIP) joint angle, proximal interphalangeal (PIP) joint angle and metacarpophalangeal (MCP) joint angle are 180°-*θ*_3_, 180°-*θ*_2_ and 180°-*θ*_1_, respectively. The length of the distal phalange, intermediate phalange, proximal phalange and metacarpal are *L*_3_, *L*_2_, *L*_1_ and *L*_0_, respectively.

The orientation and position of the finger and the fingertip are determined by the forward kinematic model and the joint angles. Here, Denavit-Hartenberg(DH) parameter approach is introduced to analyze the kinematic of the finger. Table 1 shows the DH parameters and ranges of the finger.

Based on the general form of the transformation matrix (Equation (1)) and the DH parameters (Table 1), we can achieve the transformation matrices from each phalange of the finger at its neighboring phalange as follows.

(1) Tii−1=cosθi−sinθi0ai−1sinθicosαi−1cosθicosαi−1−sinαi−1−disinαi−1sinθisinαi−1cosθisinαi−1cosαi−1dicosαi−10001

(2) T10=cosθ1−sinθ10L0sinθ1cosθ10000100001

(3) T21=cosθ2−sinθ20L1sinθ2cosθ20000100001

(4) T32=cosθ3−sinθ30L2sinθ3cosθ30000100001

The overall transformation matrix can be yielded by multiplying these three individual matrices.

(5) T30=T10T21T32=cos(θ1+θ2+θ3)−sin(θ1+θ2+θ3)0L0+L1cosθ1+L2cos(θ1+θ2)sin(θ1+θ2+θ3)cos(θ1+θ2+θ3)0L1sinθ1+L2sin(θ1+θ2)00100001

By considering the point K as the fingertip. In Figure 2, the position of the fingertip in the coordinate system of *x*_3_*y*_3_*z*_3_ is as follows.

(6) $$p_{x_{3}y_{3}z_{3}}=\left[\begin{array}{c} L_{3}\\ FK\\ 0 \end{array}\right]$$

The position of the fingertip with respect to the coordinate system of *x*_0_*y*_0_*z*_0_ can be expressed as follows.

(7) Kx0y0z0=T30L3FK01=L0+L1cosθ1+L2cos(θ1+θ2)+L3cos(θ1+θ2+θ3)−FKsin(θ1+θ2+θ3)L1sinθ1+L2sin(θ1+θ2)+L3sin(θ1+θ2+θ3)+FKcos(θ1+θ2+θ3)01

**Figure 3:**
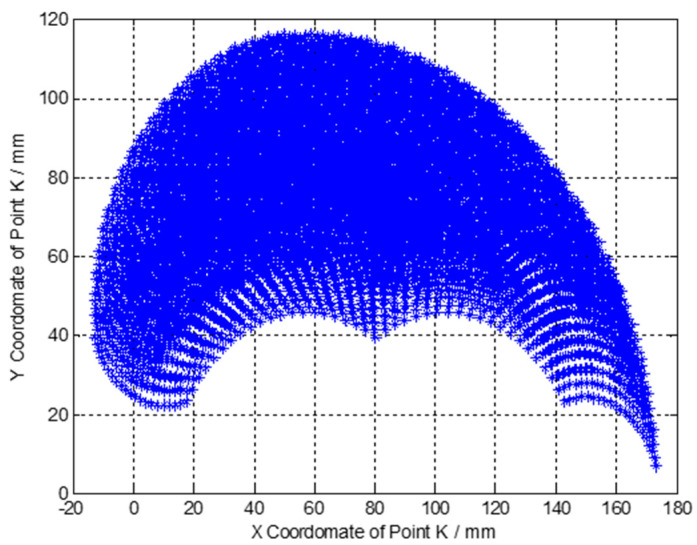
Distribution of the X and Y coordinate of the fingertip.

Based on Equation (7) and the parameters of the finger shown in Table 1, we obtained the distribution of the X and Y coordinate of the fingertip as shown in Figure 3.

#### 2.1.3. Mechanical Analysis

**Figure 4:**
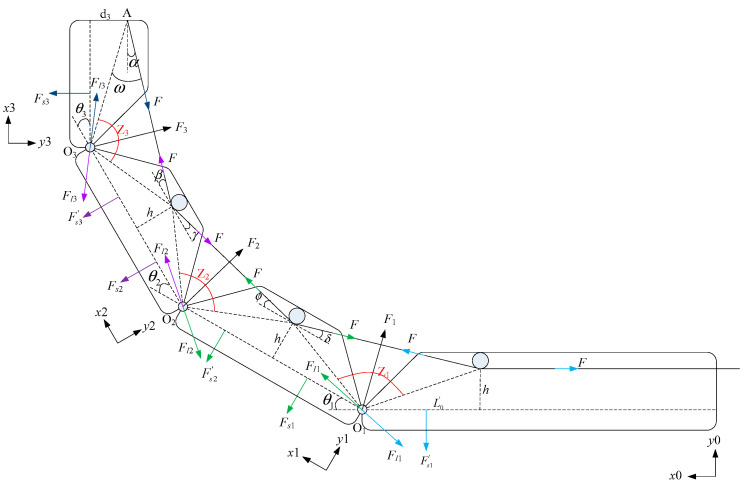
Force diagram when finger bended.

Figure 4 shows the diagram when the finger bended. The different colored arrows represent the forces exerted on the different phalanges. *F* is the driving force on the wire rope. *F_Ii_* is the force between the *i*^th^ and (*I* − 1)^th^ phalanges. *F_si_* and *F^’^_si_* are the force exerted by torsion spring on the *i*^th^ and (*I* − 1)^th^ phalanges, respectively. *F_i_* is the force of torsion spring on the shaft. Since the shaft is connected to the two adjacent phalanges by four bearings, *F_i_* is divided equally between the two phalanges. That is to say, half of *F_i_* is applied to the *i*^th^ phalange, and the other half is applied to the (*I* − 1)^th^ phalange. The relevant mechanical dimension parameters in the figure are shown in Table 2.

For the distal phalange, we can get the equilibrium equations of force and moment balance equation as follows.

(8) $$\left\{\begin{array}{l} F_{I3(x3)}+\frac{1}{2}F_{3(x3)}=F\cos\alpha \\ F_{I3(y3)}+\frac{1}{2}F_{3(y3)}+F\sin\alpha =F_{s3}\\ b_{3}F\sin\omega =F_{s3}l_{3} \end{array}\right.$$

where, *F*_*I*3(*x*3)_ and *F*_*I*3(*y*3)_ are the component of *F*_*I*3_ in the *x*3 and *y*3 directions.

Similarly, we can obtain the force balance equation and moment balance equation on the middle phalange and proximal phalange.

(9) $$\left\{\begin{array}{l} F_{I2(x2)}+F\cos\beta +\frac{1}{2}F_{2(x2)}=F_{I3(x2)}+\frac{1}{2}F_{3(x2)}+F\cos\gamma \\ F_{I2(y2)}+\frac{1}{2}F_{2(y2)}+\frac{1}{2}F_{3(y2)}+F\sin\beta +F\sin\gamma =F_{I3(y2)}+F_{s3}^{\prime }+F_{s2}\\ F_{s2}l_{2}+F_{s3}^{\prime }(L-l_{3}^{\prime })+F_{I3(y2)}L+dF\cos\beta =\frac{1}{2}F_{3(y2)}L+dF\cos\gamma +\frac{L}{2}F\sin\beta +\frac{L}{2}F\sin\gamma \end{array}\right.$$

(10) $$\left\{\begin{array}{l} F_{I1(x1)}+F\cos\phi +\frac{1}{2}F_{1(x1)}=F_{I2(x1)}+\frac{1}{2}F_{2(x1)}+F\cos\delta \\ F_{I1(y1)}+\frac{1}{2}F_{1(y1)}+\frac{1}{2}F_{2(y1)}+F\sin\delta +F\sin\phi =F_{I2(y1)}+F_{s2}^{\prime }+F_{s1}\\ F_{s1}l_{1}+F_{s2}^{\prime }(L-l_{2}^{\prime })+F_{I2(y2)}L+dF\cos\phi =\frac{1}{2}F_{2(y2)}L+dF\cos\delta +\frac{L}{2}F\sin\phi +\frac{L}{2}F\sin\delta \end{array}\right.$$

**Figure 5:**
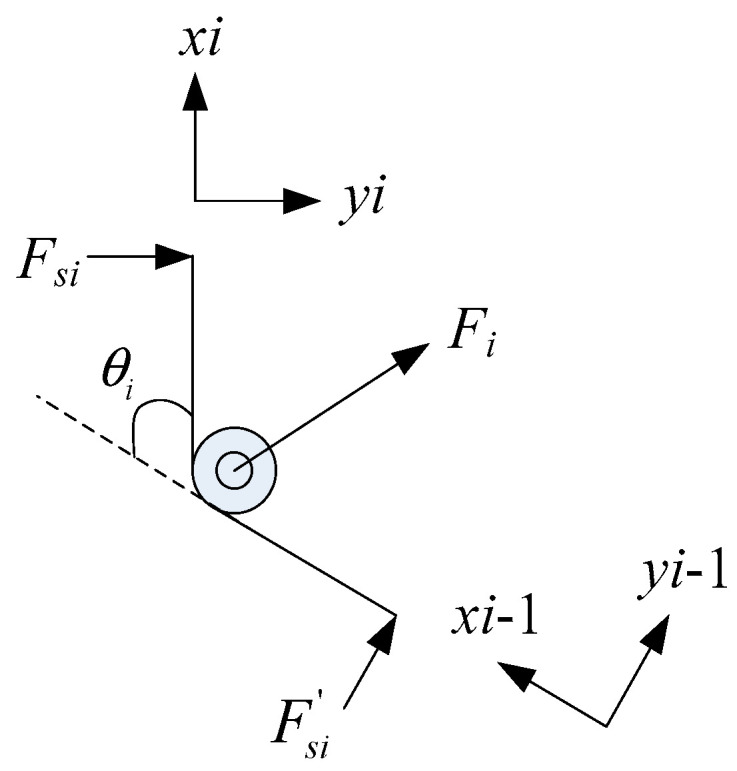
Diagram of torsion spring force analysis.

As shown in Figure 5, according to Hooke’s law, the force of *F_si_* and *F*′*_si_* can be expressed as follows.

(11) $$\left\{\begin{array}{c} F_{si}=\frac{k_{i}\theta_{i}}{l_{i}}i=1,2,3\\ F_{si}^{\prime }=\frac{k_{i}\theta_{i}}{l_{i}^{\prime }}i=1,2,3 \end{array}\right.$$

where *k_i_* is the stiffness of the torsion spring, *l_i_* and *l*′*_i_* are the length torsion spring’s legs, and *θ_i_* is the bended angle between two phalanges.

By force analysis of the torsion spring, we can know that *F_i_* is the resultant force of *F_si_* and *F*′*_si_*. The decomposition of *F_i_* in coordinate *x_i_o_i_y_i_* and *x_i−_*_1_*o_i−_*_1_*y_i−_*_1_ can be expressed as follows.

(12) $$\left\{\begin{array}{l} F_{i(xi)}=F_{si}^{\prime }\sin\theta_{i}\\ F_{i(yi)}=F_{si}+F_{si}^{\prime }\cos\theta_{i} \end{array}\right.$$

(13) $$\left\{\begin{array}{c} F_{i(xi-1)}=-F_{si}\sin\theta_{i}\\ F_{i(yi-1)}=F_{si}^{\prime }+F_{si}\cos\theta_{i} \end{array}\right.$$

For the interaction, *F_Ii_*, between the two phalanges, we can get the following relationship.

(14) $$\left\{\begin{array}{c} F_{Ii}{}_{\left(yi\right)}\sin\theta_{i}+F_{Ii}{}_{\left(xi\right)}\cos\theta_{i}=F_{Ii(xi-1)}\\ F_{Ii}{}_{\left(yi\right)}\cos\theta_{i}-F_{Ii}{}_{\left(xi\right)}\sin\theta_{i}=F_{Ii(yi-1)} \end{array}\right.$$

where, *F_Ii(xi)_* and *F_Ii(yi)_* are the components of *F_Ii_* in the *xi* and *yi* directions, and *F_Ii(xi−_*_1*)*_ and *F_Ii(yi−_*_1*)*_ are the components of *F_Ii_* in the *x_i_*_−1_ and *yi*-1 directions.

For the angle between the wire rope and phalanges, *α*, *β*, *γ*, *δ* and *θ*, we can obtain the following relationship according to the law of sines.

(15) $$\alpha =\arcsin\left(\frac{b_{3}}{c_{3}}\cdot \sin z_{3}\right)-\arctan\frac{d_{3}}{L_{1}}\;\beta =\arcsin\left(\frac{a_{3}}{c_{3}}\cdot \sin z_{3}\right)-\arctan\frac{h}{L_{2}}\;\gamma =\arcsin\left(\frac{b_{2}}{c_{2}}\cdot \sin z_{2}\right)-\arctan\frac{h}{L_{3}}\;\phi =\arcsin\left(\frac{a_{2}}{c_{2}}\cdot \sin z_{2}\right)-\arctan\frac{h}{L_{2}}\;\delta =\arcsin\left(\frac{b_{1}}{c_{1}}\cdot \sin z_{1}\right)-\arctan\frac{h}{L_{3}}$$

where,

(16) $$z_{3}=180^{\circ }-\arctan\frac{d_{3}}{L_{3}}-\arctan\frac{h}{\frac{1}{2}L_{2}}-\theta_{3}\;z_{2}=180^{\circ }-\arctan\frac{h}{\frac{1}{2}L_{2}}-\arctan\frac{h}{\frac{1}{2}L_{1}}-\theta_{2}\;z_{1}=180^{\circ }-\arctan\frac{h}{\frac{1}{2}L_{1}}-\arctan\frac{h}{L_{0}^{\prime }}-\theta_{1}$$

The following relation can be obtained by connecting the above Equations (8)–(16).

(17) $$\begin{array}{ll} \{ & \begin{array}{ll} k_{1}\cdot \theta_{1}= & \frac{L_{1}}{2}\cdot \left(\frac{k_{2}\cdot \theta_{2}}{l_{2}^{\prime }}+\frac{k_{2}\cdot \theta_{2}}{l_{2}}\cdot \cos\theta_{2}\right)-\left(F_{I2\_x2}\cdot \sin\theta_{2}+F_{I2\_y2}\cdot \cos\theta_{2}\right)\cdot L_{1}\\ & +\frac{L_{1}}{2}\left(F\cdot \sin\phi +F\cdot \sin\delta \right)+\left(F\cdot \cos\phi -F\cdot \cos\delta \right)\cdot h-\frac{1}{2}\cdot \frac{k2\cdot \theta 2}{l_{2}^{\prime }}\cdot \left(L_{1}-l_{2}^{\prime }\right)\\ k_{2}\cdot \theta_{2}= & \frac{L_{2}}{2}\cdot \left(\frac{k3\cdot \theta 3}{l_{3}^{\prime }}+\frac{k3\cdot \theta 3}{l_{3}}\cos\theta 3\right)+\frac{L_{2}}{2}\cdot \left(F\cdot \sin\beta +F\cdot \sin\gamma \right)\\ & +(F\cdot \cos\gamma -F\cdot \cos\beta )\cdot h-\left(F_{I3\_x3}\cdot \sin\theta_{3}+F_{I3\_y3}\cdot \cos\theta_{3}\right)\cdot L_{2}-\frac{k3\cdot \theta 3}{l_{3}^{\prime }}\cdot \left(L_{2}-l_{3}^{\prime }\right)\\ k_{3}\cdot \theta_{3}= & \frac{F\cdot b_{3}\cdot a_{3}}{c_{3}}\cdot \sin z_{3}\\ F_{I1\_x1}= & F\cdot \cos\phi +F_{I2\_x2}\cdot \cos\theta_{2}-F_{I2\_y2}\cdot \sin\theta_{2}\\ & +\frac{1}{2}\cdot \frac{k_{2}\cdot \theta_{2}}{l_{2}}\cdot \sin\theta_{2}-\frac{1}{2}\cdot \frac{k_{1}\cdot \theta_{1}}{l_{1}^{\prime }}\cdot \sin\theta_{1}-F\cdot \cos\delta \\ F_{I1\_y1}= & \frac{1}{2}\cdot \frac{k_{2}\cdot \theta_{2}}{l_{2}^{\prime }}+\frac{1}{2}\cdot \frac{k_{2}\cdot \theta_{1}}{l_{1}}-\frac{1}{2}\cdot \frac{k_{2}\cdot \theta_{2}}{l_{2}}\cos\theta_{2}-F\cdot \sin\delta -F\cdot \sin\phi \\ & +F_{I2\_x2}\cdot \sin\theta_{2}+F_{I2\_y2}\cdot \sin\theta_{2}-\frac{1}{2}\cdot \frac{k_{1}\cdot \theta_{1}}{l_{1}^{\prime }}\cdot \cos\theta_{1}\\ F_{I2\_x2}= & F\cdot \cos\gamma +\frac{1}{2}\cdot \frac{k_{3}\cdot \theta_{3}}{l_{3}}\cdot \sin\theta_{3}+F_{I3\_x3}\cdot \cos\theta_{3}\\ & -\frac{1}{2}\cdot \frac{k2\cdot \theta 2}{l_{2}^{\prime }}\cdot \sin\theta_{2}-F\cdot \cos\beta -F_{I3\_y3}\cdot \sin\theta_{3}\\ F_{I2\_y2}= & \frac{1}{2}\cdot \frac{k_{3}\cdot \theta_{3}}{l_{3}^{\prime }}+\frac{1}{2}\cdot \frac{k2\cdot \theta 2}{l_{2}^{\prime }}+F_{I3\_x3}\cdot \sin\theta_{3}+F_{I3\_y3}\cdot \cos\theta_{3}-F\cdot \sin\gamma \\ & -F\cdot \cos\beta -\frac{1}{2}\cdot \frac{k_{2}\cdot \theta_{2}}{l_{2}}-\frac{1}{2}\cdot \frac{k_{2}\cdot \theta_{2}}{l_{2}^{\prime }}\cdot \cos\theta_{2}-\frac{1}{2}\cdot \frac{k_{3}\cdot \theta_{3}}{l_{3}}\cdot \cos\theta_{3}\\ F_{I3\_x3}= & F\cdot \cos\alpha -\frac{1}{2}\cdot \frac{k_{3}\cdot \theta_{3}}{l_{3}^{\prime }}\cdot \sin\theta_{3}\\ F_{I3\_y3}= & \frac{1}{2}\cdot \left(\frac{k_{3}\cdot \theta_{3}}{l_{3}}-\frac{k_{3}\cdot \theta_{3}}{l_{3}^{\prime }}\cdot \cos\theta_{3}\right)-F\cdot \sin\alpha \end{array} \end{array}$$

In the structure of the finger proposed in this paper, the torsion spring embedded in the finger joint is an important factor affecting the motion law of the bionic finger. Torsion springs with different stiffness coefficient lead to different motion characteristic of the finger. And the following three types of movements may occur in the procedure of the finger grasping an object.

**Figure 6:**
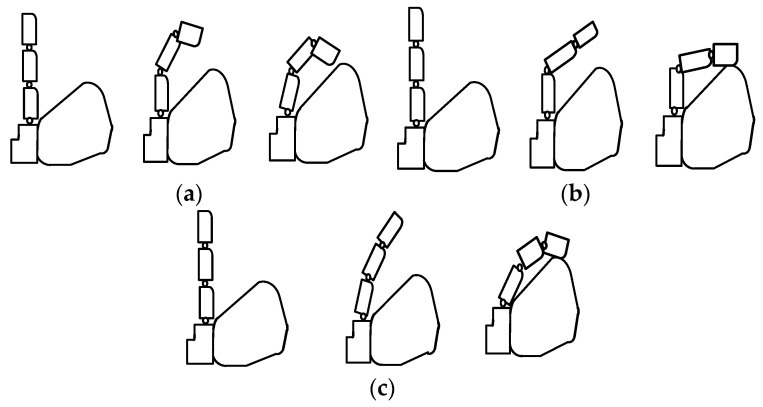
Sketch map of finger movement law. (**a**) The first type of the movement; (**b**) The second type of the movement; (**c**) The third type of the movement.

One case is that the small stiffness coefficient of torsion spring implanted in DIP joint leads to the asynchronous movement of each joint of the bionic finger. And the DIP joint bends at an angle greater than the MCP and PIP joints. The movement process is shown in Figure 6a. The other case is that the small stiffness coefficient of torsion spring implanted in PIP joint also leads to the asynchronous movement of the finger. In extreme cases, the proximal finger joint hardly moves until it touches the object, while the distal finger joint can only bend relatively slightly. The movement process is shown in Figure 6b. There is another case that the small stiffness coefficient of torsion spring implanted in MCP joint will cause both PIP and DIP joints to have almost no relative bending movement during the whole movement process. In this case, the three phalanges of the bionic finger seem to be equivalent to one phalange, which makes the bionic hand lack dexterity.

Obviously, the finger motion laws discussed above are not suitable for grasping objects. However, if the stiffness coefficient of the torsion spring at each joint of the finger is selected properly, the bionic finger can achieve an adaptive motion rule similar to that of a human hand, which makes the bionic finger have good grasping performance. This finger motion schematic is shown in Figure 6c. In this case, the bionic finger can adapt to objects of different shapes and sizes to achieve stable grasping.

**Figure 7:**
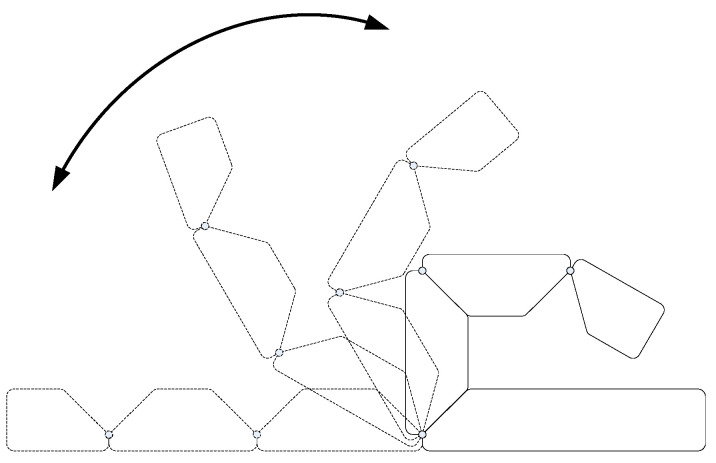
Finger flexion showing the desired trajectory.

During natural movement, the movement of the finger is similar to that shown in Figure 6c. Each joint of the finger moves synchronously and the rotation angle of each joint satisfies a certain movement law [13]. In order to simplify the design of bionic finger structure parameters and realize the adaptive motion as shown in Figure 6c, the motion relationship of joint rotation is simplified here. As shown in Figure 7, the rotation angles of PIP, DIP and MCP joints approximately satisfy the relationship of 3:3:1. Meanwhile, it is considered that the maximum bending angle of each joint in the finger designed in this paper is 90°. Therefore, in the selection of spring parameters *θ*_1_ = *θ*_2_ = 3*θ*_3_ = 90° is taken as a constraint.

In this paper, the *K*_1_ and *K*_2_ parameters are obtained as follows on the premise of choosing *K*_3_ = 2.8 N·mm/degree based on the limiting relation of rotation angles of the three joints mentioned above.

(18) $$\left\{\begin{array}{c} K_{1}=1.5531\;N\cdot \mathrm{mm}/\mathrm{degree}\\ K_{2}=1.4119\;N\cdot \mathrm{mm}/\mathrm{degree} \end{array}\right.$$

In practice, we choose torsion springs with similar stiffness coefficients in the market. The selected torsion springs with the stiffness are as follows.

(19) $$\left\{\begin{array}{c} K_{1}=1.605\;N\cdot \mathrm{mm}/\mathrm{degree}\\ K_{2}=1.375\;N\cdot \mathrm{mm}/\mathrm{degree} \end{array}\right.$$

### 2.2. Mechanical-Sensor Integrated Finger Joint

The capacity of information perception is important for a dexterous hand. There are many kinds of thin-film force or tactile sensors that can be easily assembled on the surface of a bionic finger to capture information such as grasp force. However, joint angles are much more difficult to obtain because there is usually not enough space on the finger for angle sensors. This subsection describes in detail the design of a mechanical sensor-integrated finger joint, which mainly consists of two parts: analysis of finger joint structure and signal measurement circuit. The design is to achieve accurate measurement of the bending angle of each joint when grasping an object.

#### 2.2.1. Analysis of Finger Joint Structure

**Figure 8:**
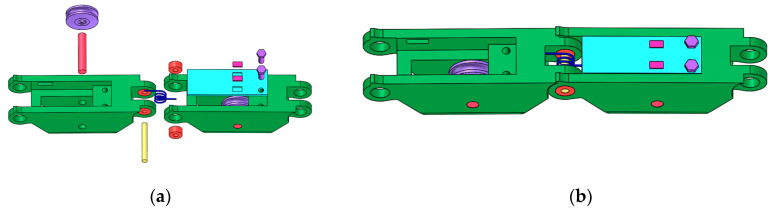
Assembly drawing of the finger joint. (**a**) Components of the finger joint; (**b**) Finger joint.

Figure 8 shows a designed finger joint. The rotatable joint consists of a roller, a torsion spring and four bearings. The roller passes through four bearings, a torsion spring and two phalanges. One leg of the torsion spring is fastened to one phalange, and the other leg is pressed by a small aluminum plate. The aluminum plate is fastened to the other phalange with two screws.

Take the joint between proximal phalange and metacarpal for example. The torsion spring will output a torque when there is an angle between the proximal phalange and metacarpal. Because two legs of the torsion spring are restricted by the aluminum plate and the phalange, the torque output from the spring will be converted to force and be applied to the aluminum plate.

**Figure 9:**
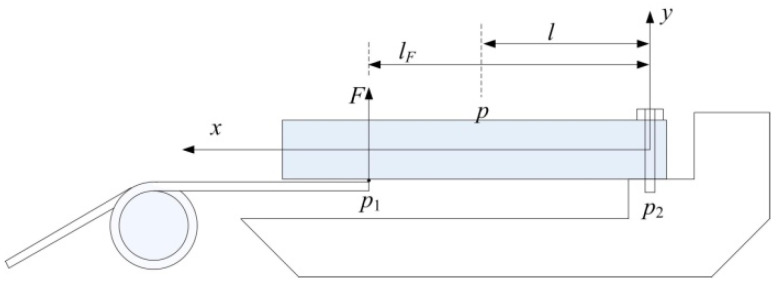
The simplified assembly drawing of the aluminum plate and the torsion spring.

The aluminum plate can be considered as a cantilever beam because one side of the aluminum plate is fixed on the metacarpal bone by two screws. A simplified assembly drawing for aluminum plates and torsion springs has been shown in Figure 9. *l_F_* is the distance between the point *p*_1_ and *p*_2_. *p*_1_ is the contact position of the torsion spring and the aluminum plate. *p*_2_ is the root of the aluminum plate.

When a finger bends, the axial strain at point *p*, on the surface of the aluminum plate is as follows:

(20) $$\varepsilon_{p}=\frac{6F}{Ebh^{2}}(l_{F}-l)$$

where *E* is the Young’s modulus of the material, *l* is the distance between point *p* and *p*_2_, *b* and *h* are the width and thickness of the cantilever beam respectively

Combining Equations (11) and (20), we can get the relationship between finger joint angle (*θ*) and axial strain on the surface of the aluminum plate ($\varepsilon_{P}$) as follows:

(21) $$\theta =\varepsilon_{P}\frac{Ebh^{2}}{6(l_{F}-l)kl_{s}}$$

Where *θ* and $\varepsilon_{P}$ are linearly related in the recoverable deformation range of the aluminum plate. In other words, as long as we can get $\varepsilon_{P}$, the finger joint angle(*θ*) can be obtained by simple calculation.

#### 2.2.2. Signal Measurement Circuit

A strain gauge is used to measure the axial strain on the surface of the aluminum plate in this paper. Within the limit of the strain gauge, the relationship between the axial strain and the variation of the resistance is linear.

The maximum bending angle of the designed finger in this paper is 90°. Therefore, the maximum axial strain on the surface of the aluminum plate can be described as follows.

(22) $$\varepsilon_{{}_{P\_\max}}=\frac{kl_{s}\theta_{\max}6(l_{F}-l)}{bh^{2}E}=\frac{540kl_{s}(l_{F}-l)}{bh^{2}E}$$

Based on the above analysis, the strain gauges with the parameters shown in Table 3 are used to achieve the measurement of the axial strain on the surface of the aluminum plate.

**Figure 10:**
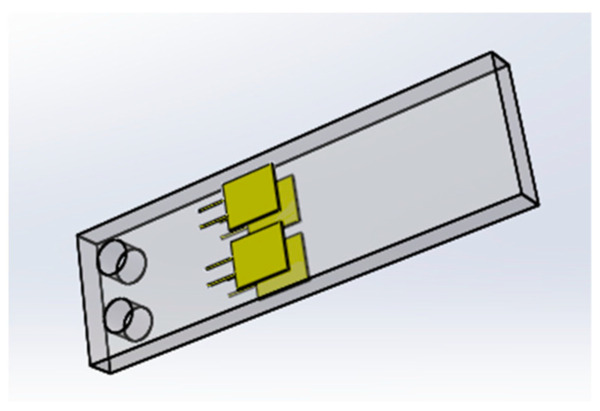
Stick diagram of strain gauges.

As shown in Figure 10, four strain gauges were pasted on the upper and the lower surface of the aluminum plate respectively. Among of them, two strain gauges sg_2_ and sg_3_, were pasted on the upper surface of the aluminum plate, and the other two strain gauges, sg_1_ and sg_4_, were pasted on the lower surface. When the fingers are bent, the force exerted by the torsion spring on the aluminum plate causes deformation of the plate. And the deformation of the aluminum plate will likewise cause the deformation of the strain gauge. Since the extensional deformation and compressive deformation lead to the increase and decrease of strain gauge resistance respectively, the finger bending leads to the increase of resistance of sg_1_ and sg_4_ and the decrease of resistance of sg_2_ and sg_3_. And since sg_2_, sg_3_ and sg_1_, sg_4_ are distributed symmetrically, the resistance variations of sg_1_, sg_2_, sg_3_ and sg_4_ are the same.

**Figure 11:**
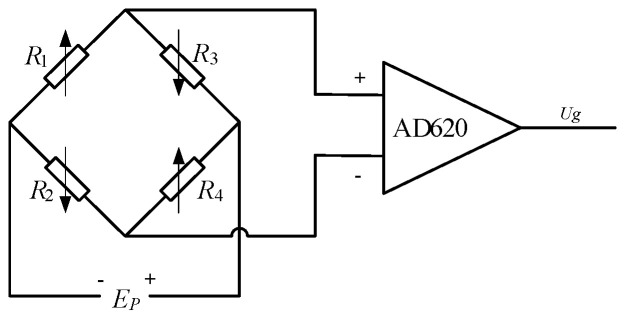
Measurement circuit of the strain gauges.

As shown in Figure 11, the strain gauges, sg_1_, sg_2_, sg_3_ and sg_4_, are plugged into the circuit. Amplifier based on AD620 is used to amplify the output signal of the Wheatstone bridge. Under the assumption that sg_1_ and sg_3_ are pasted on the surface of the cantilevered beam symmetrically and *R*_1_ = *R*_2_ = *R*_3_ = *R*_4_, we can get the output of the circuit:

(23) $$U_{g}=A\frac{\Delta R_{1}}{R_{1}}E_{P}$$

where *E_P_* is the value of the supply power, △*R*_1_ is resistance variation of the four strain gauges, *A* is the amplification.

The relationship between strain and relative variation of the resistance, $\frac{\Delta R}{R}=K\varepsilon$, is linear within a large range. The relationship between the circuit output and the strain of the strain gauge can be expressed as follows.

(24) $$\theta =\frac{F}{kl_{s}}=\frac{Ebh^{2}}{6(l_{F}-l)AE_{P}K_{}kl_{s}}U$$

where *K* is the sensitivity factor of the strain gauge.

There are three finger joints with the same structure in the designed finger. Therefore, three measurement circuits are designed as shown in Figure 11.

**Figure 12:**
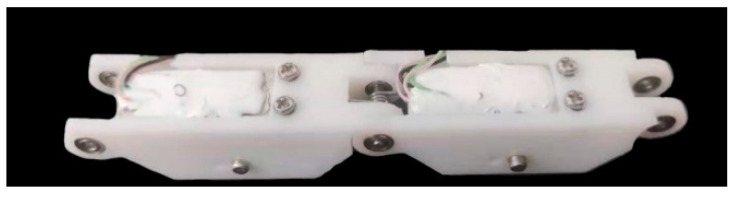
Phalanges equipped with aluminum plates.

Figure 12 shows the phalanges equipped with aluminum plates.

## 3. Experiments and Results

To evaluate the designed finger, joint angle measurement experiment, movement law evaluation experiment and objects’ grasping experiment are conducted.

### 3.1. Joint Angle Measurement Experiment

The experimental setup consists of a finger, a steering gear, three gyroscopes, a data acquisition card and computer software.

**Figure 13:**
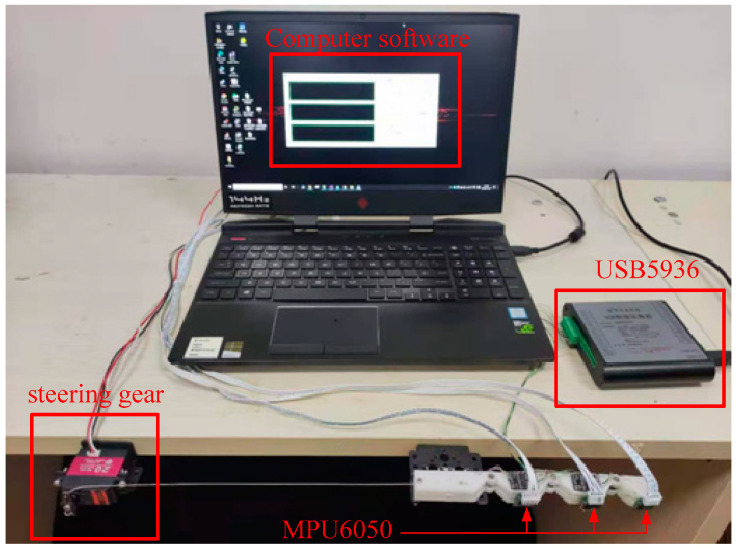
Joint angle measurement.

As shown in Figure 13, a finger and a steering gear are fixed horizontally on the edge of the table. The wire rope is fixed to the swing arm of the steering gear. Finger bending can be realized by controlling the swinging of the steering gear. Three six-axis gyroscopes based on MPU6050 are employed to measure angles of the finger joints. The gyroscopes are pasted on the surface of the phalanges. The gyroscope outputs the rotation angle in real time at a speed of 100Hz. The dynamic measurement precision is 0.1°. A data acquisition card (USB5936) is employed to capture the signals output from the strain gauge measurement circuits. And the digital signals are sent to computer via USB. Computer software based on MFC framework is developed to display the signals and record the data.

**Figure 14:**
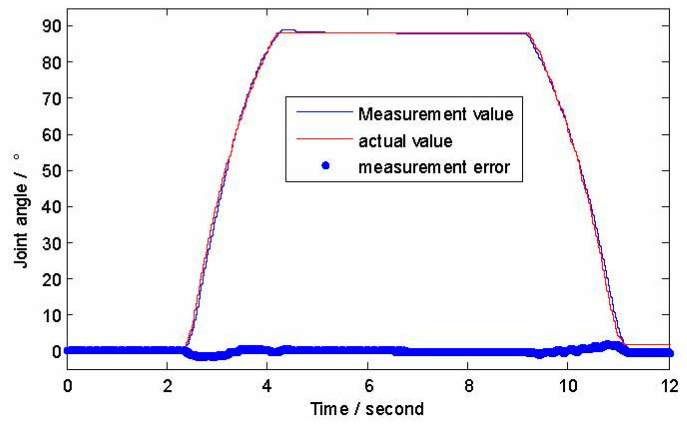
The results of the finger joint angle measurement experiment.

Taking MCP angle measurement for example, the steering gear is rotated so that the proximal phalange gradually bends from 0° to 90°, and then gradually recovers from 90° to 0°. At the same time, the signal output from the gyroscope is synchronously recorded with the strain gauge measurement circuit. Figure 14 shows experimental results of the MCP angle measurement. The RMS of measurement errors of the DIP, PIP and MCP are 0.36°, 0.59° and 0.32°, respectively.

### 3.2. Evaluating the Relationship between Driving Force and Finger Bending Angle

**Figure 15:**
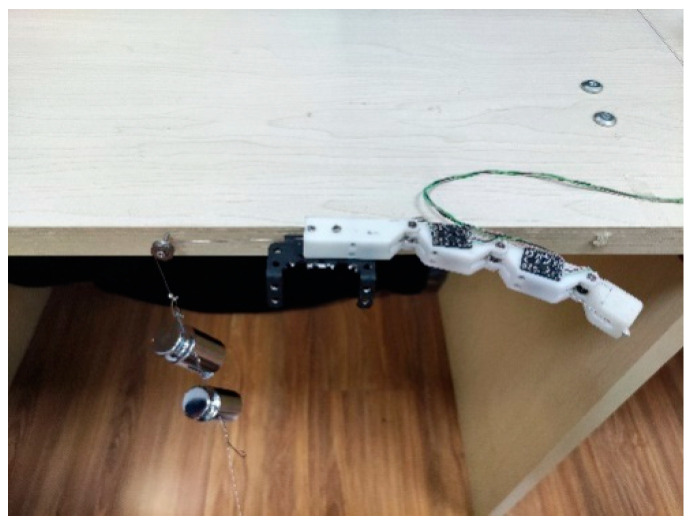
Experimental setup for evaluating the relationship between driving force and finger bending angle.

Figure 15 shows an image of the experiment, where the finger is fixed to the side of the table and a weight draws the rope through a pulley to rotate the finger joints.

**Figure 16:**
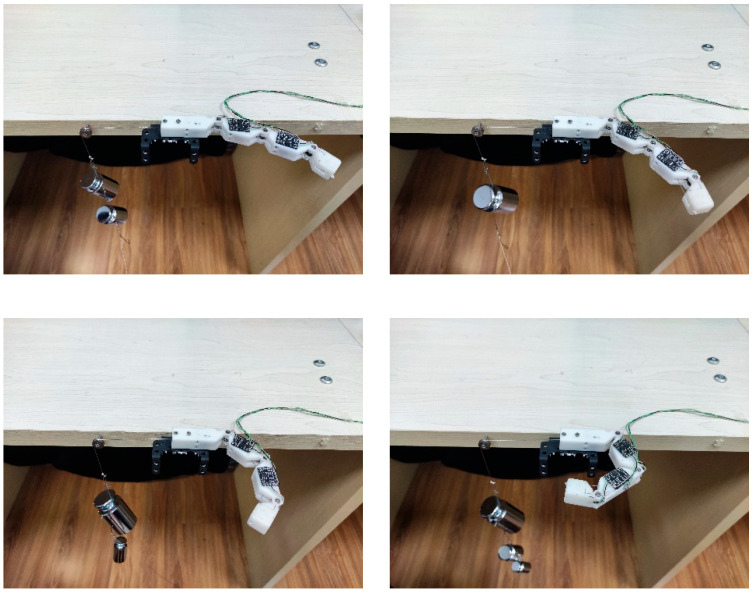
A movement of the finger as the driving force is gradually increased.

**Figure 17:**
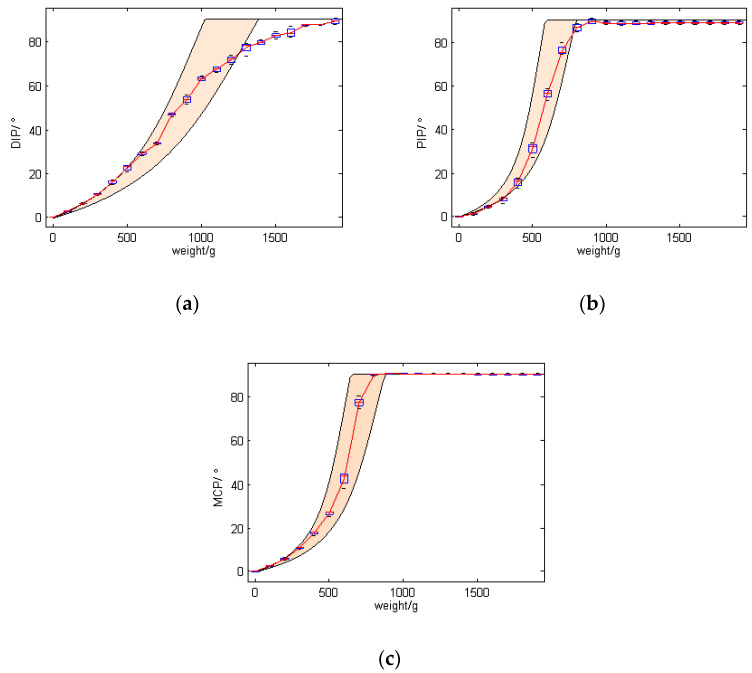
Relationship between driving force and finger joints’ angle. (**a**) The results of DIP joint; (**b**) The results of PIP joint; (**c**) The results of MCP joint.

In this experiment, 18 weights from 100 g to 1800 g with increments of 100 g were used to test the relationship between driving force and finger bending angle for 4 times. Figure 16 shows the movement of the finger as the driving force gradually in-creases, and Figure 17 shows the bending angles of the finger joints with different driving forces. The bending angles of PIP and MCP joints are basically consistent throughout the movement. For the weights from 100 g to 800 g, the angles of all finger joints increase with increasing driving force. It basically satisfies the relationship θ1 = θ2 = 3θ3. The angles of PIP and MCP reached a maximum bending angle of 90° when the driving force was around 800 g. Consequently, from 800 g to 1800 g, the angles of PIP and MCP remained the same, while the angle of the DIP joint increased as the driving force increased. These experimental results have an important role in the con-trol of the finger in a later stage.

Considering the spring stiffness coefficient with ±15% error, the distribution area of the joints’ angle on different driving force is fulfilled with peach puff as shown in Figure 17. As can be seen from the figure, bending angles of the finger joints conforms to the design expectation.

### 3.3. Evaluating the Relationship between Displacement of the Driving Cable and Finger Joints’ Angle

**Figure 18:**
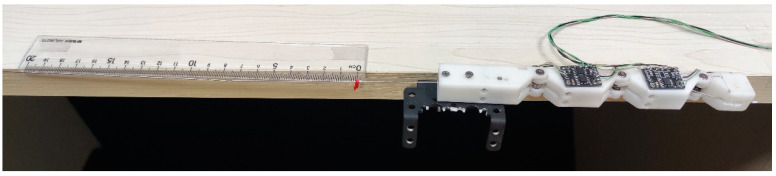
Experimental setup for evaluating the relationship between displacement of the driving cable and finger joints’ angle.

As in the previous experiment, the finger is fixed to the side of the table. A red light pointer is tied to the wire rope. Along with the ruler placed on the edge of the table, the displacement of the wire rope can be displayed. The image of the experiment is shown in Figure 18.

**Figure 19:**
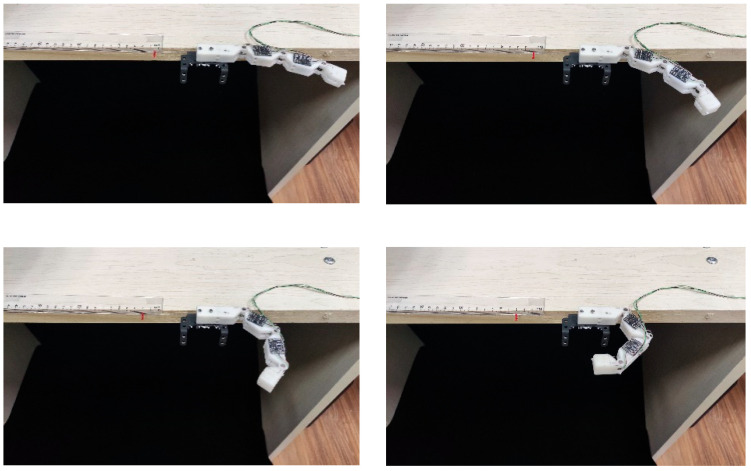
A movement of the finger as the displacement of driving rope is gradually increased.

**Figure 20:**
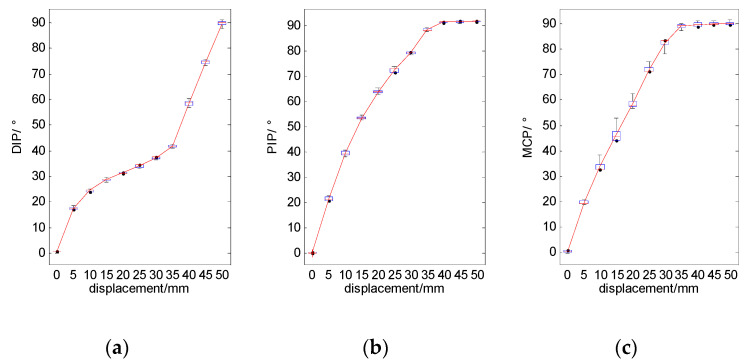
Relationship between displacement of driving rope and finger joints’ angle. (**a**) The results of DIP joint; (**b**) The results of PIP joint; (**c**) The results of MCP joint.

We measured the joint angle of the finger with increments of 5 mm within the displacement range from 0 to 50 mm. Figure 19 shows a movement of the finger as the displacement of the driving cable gradually increases. And Figure 20 shows the angle of the finger joints under different displacement of the driving cable.

For the displacement from 0 mm to 35 mm, the angles of all finger joints increase with the increase of driving force. The angles of PIP and MCP reach a maximum bending angle of 90° when the driving force is around 35 mm. Consequently, from 35 mm to 50 mm, the angle of DIP joint increases as the driving force increases.

The results of this experiment also showed that the motion of the fingers was consistent with the design expectation.

### 3.4. Objects’ Grasping Experiment

**Figure 21:**
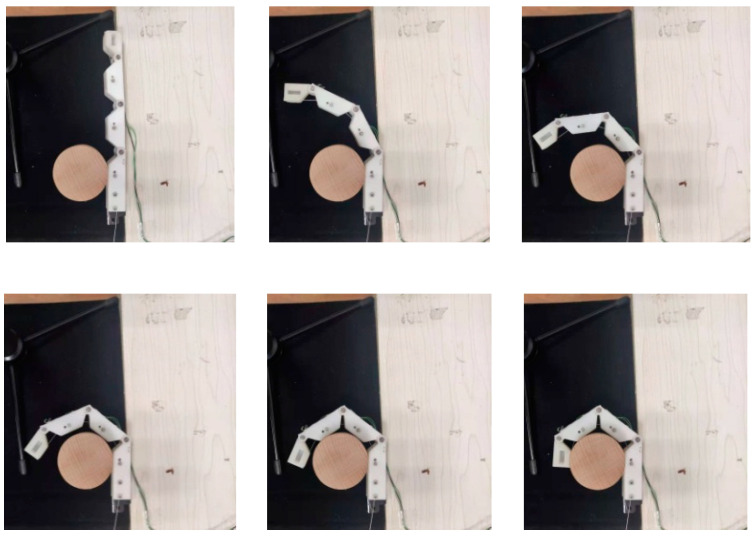
Adaptive grasping process of a cylinder.

**Figure 22:**
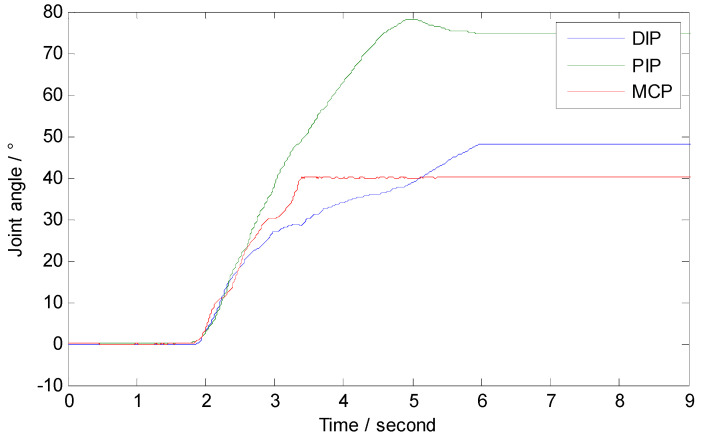
The joints’ angle during grasping a cylinder.

**Figure 23:**
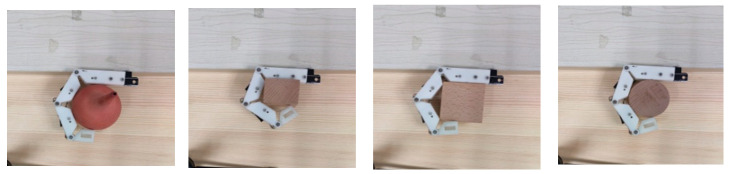
Different objects grasping scene by the designed finger.

To verify that the designed fingers can adapt to objects of different shapes and can grasp them, four objects with different shapes, spherical, cuboid, cube, and cylinder, were selected for the experiment. Take the grasping of a cylinder for example. As shown in Figure 21, when the finger was in the state of unbending, the cylinder was place on the inside of the finger and then the finger was controlled to gradually bend and fully grasp and hold the cylinder through the steering gear. The angles of the finger joints during this process are shown in Figure 22. In the experiment, the proximal phalange contacted the cylinder first, followed by the intermediate phalange and finally the distal phalange. The results in Figure 22 show that, when the proximal phalange is in contact with the cylinder, the angle of the MCP hardly changes because movement of the phalange is restricted by the cylinder. The movements of the distal phalange and intermediate phalange are the same as that of the proximal phalange. Because of this movement law, the designed finger can adapt to objects with different shapes. Figure 23 shows stable grasping of objects with different shapes.

## 4. Conclusions

In order to achieve dexterous manipulation of a robot hand, an adaptive finger with joint angle measurement capability is proposed in this paper. The proposed finger has three joints. The mechanical composition of the designed finger is described in detail. Forward kinematics is conducted to analyze the distribution of the fingertip. Moreover, on the basis of force analysis of each phalanx, a set of spring coefficients is selected to make the relationship among MCP, PIP and DIP approximately satisfy 3:3:1 when the finger moves in free space. In addition, the composition, the principle and the measurement circuit of the mechanical-sensor integrated finger joint are described in detail. In the experimental section, joint angle testing, movement law evaluation and objects grasping experiments are conducted to verify the designed finger. The experimental results show that the designed finger can achieve the measurement of the joint angle of the finger with the RMS of measurement errors of MCP, PIP and DIP are 0.32°, 0.59° and 0.36°, respectively. The bending angles of the finger joints conform to the design requirements when the finger moves in free space. And the objects of different shapes can be stably grasped with the designed finger.

For the next step, we will study the control strategy for the designed finger and design a hand with the finger proposed in this paper.

## Figures and Tables

**Table 1:** Parameters and ranges of the links.

*i*	*a*_*i*−1_	*α*_*i*−1_	*d_i_*	*θ_i_*	*Range*
0	0	0	0	0	
1	*L*_0_	0	0	*θ*_1_	0–*π*/2
2	*L*_1_	0	0	*θ*_2_	0–*π*/2
3	*L*_2_	0	0	*θ*_3_	0–*π*/2

**Table 2:** Mechanical dimension parameters of the phalanges.

Parameter	Value (mm)	Description
*L*_0_	57	Length of the *i*^th^ phalange, *i* = 0, 1, 2 and 3 represents the metacarpal, proximal phalange, intermediate phalange and distal phalange, respectively.
*L*_1_	46.5	
*L*_2_	46.5	
*L*_3_	23.25	
$l_{1}$	5	Length of the torsion springs’ leg. *l_i_* represents the length of spring’s leg on the side of the *i*^th^ phalange. $l_{1}^{\prime }$ represents the length of spring’s leg on the side of the (*i* − 1)^th^ phalange.
$l_{1}^{\prime }$	5	
$l_{2}$	5	
$l_{2}^{\prime }$	5	
$l_{3}$	4	
$l_{3}^{\prime }$	5	
$L_{0}^{\prime }$	23.25	In the *x*_0_ direction, the distance between the pulley and the shaft.
*h*	3	In the *y* direction of each phalange, the distance between the pulley and the shaft.
*d*_3_	10	In the *y*_3_ direction, the distance between the wire rope fixed point and the shaft.

**Table 3:** Parameters of the strain gauge.

Parameter	Value
Length (mm)	3.6 mm
Width (mm)	3.1 mm
Resistance (Ω)	350
Sensitivity factor	2.0 ± 1%
Strain limit (um/m)	20,000

## Data Availability

The data presented in this study are available in article.
